# Progress in Objective Detection of Depression and Online Monitoring of Patients Based on Physiological Complexity

**DOI:** 10.3389/fpsyt.2022.828773

**Published:** 2022-03-28

**Authors:** Milena Čukić, Victoria López

**Affiliations:** ^1^Institute for Technology of Knowledge, Complutense University, Madrid, Spain; ^2^3EGA B.V., Amsterdam, Netherlands; ^3^General Physiology and Biophysics Department, Belgrade University, Belgrade, Serbia; ^4^Quantitative Methods Department, Cunef University, Madrid, Spain

**Keywords:** detection, monitoring, depression, machine learning, physiological complexity

The advent of artificial intelligence (AI) and machine learning (ML) in particular, in medicine, holds many promises. Although the acceptance of any innovation in medicine is chronically slow, psychiatry showed to be especially conservative in this regard (1). There are brilliant examples of ML applications in medicine, such as skin-cancer/sarcoma detection (2), early detection of retinopathies (3), and many more (4). But despite a lot of effort invested in computational psychiatry projects (5) we can see zero clinical applications (6). In addition, recent publications coming from review done by AI experts are showing that ‘medical information is more complex and less available than the web data that many algorithms were trained on, so results can be misleading' (7, 8).

Our group focused mainly on data-driven computational psychiatry research (9–14). We also became aware of so-called unwarranted optimism (15–17) and reported on it (10, 12). The expression' unwarranted optimism' is coined in ML community to signify for unrealistically inflated high accuracies of models due to unresolved Dimensionality of problem, absent external validation, unproportional ratio between number of variables and number of subjects in high-dimensional medical datasets, and existance of unattended blind spots. It also illustrates the phenomenon that the scientific community, in our opinion, lulles itself into thinking that we are developing models that work much better than they actually do (7).

A recent publication demonstrated that ML's purely reliance on patient's medical history, medication, epidemiological data, and scales/questionaries data (18) are simply not capable of providing practically useful results. We also explored the possibilities of this methodology in forecasting mania in bipolar depression disorder-BDD (13, 14, 19, 20). In this research we collected daily self-reports (via mobile phone applications), clinical assessment (standard clinical interviews and scales/questionaries), medical histories (including medication, and other important variables), several sleep variables, smartwatch variables (173 variables per person in total) in attempt to construct an accurate dynamical model of transition between five clinically defined states and in order to forecast mania phase. We used complex pipeline, several feature extraction methods, several feature selection models, and applied four different ML models and network flow model, in order to mathematically describe clinically compiled data (to represent the bidirectional transition between five distinct phases in BDD). The aim was in essence to extract the most relatable variables that have prognostic value in early warning of mania, that resulted in real personalized medicine application. Among all the variables the best predictors of mania were sleep quality (and duration) and irritability (13), and Random Forest scored the best. The classification using only selected variables produced better results than using all available information. Hence, dimensionality reduction of a problem was crucial to this research.

Whelan, Garavan, Gillan, and their colleagues, explained in their publications before 2017, why computational psychiatry projects, even when relying on neuroimaging data are flawed (16, 17), arguing that some basic postulates from Information theory and Statistical learning theory are ignored, despite wide accessibility of many ML models. The consequence is overly optimistic (and misleading) results, that are not leading to clinically useful applications [see also (21–23)]. There are many publications that confirmed (among them the 2021 report from Alan Turing Institute on faulty AI application in Health) the notion that majority of AI applications in Health are simply yielding very poor results, like for example famous IBM's Watson for Oncology that failed catastrophically [(24) report (25)]. See for example (7, 26–28), for review of this particularly inflated expectations of machine learning applications in Health. As phenomenon described in Statistical Learning Theory, a “Curse of dimensionality”, demonstrated to be the central problem in particular with datasets with the large number of features in vast digital health data, shown to be challenging the development of robust AI models (in particular, their generalizability). Whenever you sample from all the possible values, the average interpoint distance between samples is rising as the dimensionality of that data space changes (1D, 2D, 3D, etc.). The increased sparsity in the relevant feature space exponentially increases the volume of blind spots in data (7). Those are contiguous regions of feature space for which we don't have samples. By this the training set becomes biased in an important way, and so fails to include samples from the region (7). A small high-dimensional training sample (characteristic for majority of health applications) is susceptible to dataset blind spots (26). Also, the volume of blind spots scales exponentially with the number of features. If data from the sample is susceptible to blind spots and the data from those blind spots are encountered *after* deployment, the model can produce incorrect treatment recommendations that are not detected during model development (7).

We argue here that the central thing that *can* lead to the resolution of this frustration, is an addition of electrophysiological signals analysis, and appropriate characterization of it which yields highly accurate results in detection and prediction of any ML model used (11, 12). The overall accuracies per seven ML models used, depending of the number of principal components included, were between 92 and 95%, showing that the *proper non-linear characterization* of a resting EEG was the key for practically useful detection. We showed that in this way (characterizing EEG with non-linear measures capable to accurately detect its intrinsic dynamics), it is possible to discern between episode and remission phase in MDD (9), besides accurate detection of depression. Other groups of researchers demonstrated that it is possible to detect who is the responder to transcranial magnetic stimulation (rTMS), since this therapy has repeatedly been shown to be effective even in treatment resistant depression (TRD) (29, 30). Another non-invasive brain stimulation technique (NIBS), transcranial direct current stimulation (tDCS), has shown to be effective in MDD treatment (31, 32). We demonstrated how this modality of stimulation leaves a detectable impact on the brain lasting longer than half an hour after the stimulus was presented (9). In another publication, we explained why NIBS techniques might work in depression treatment, based on the physiological complexity approach (32). By connecting earlier findings coming from fMRI research (33), observed increased complexity in EEG (34, 35) and already mentioned decreased complexity after the therapy (29, 30), we concluded that the feature of successful therapy for depression, must be connected to its ability to decrease said aberrated complexity, that represents the distinct internal dynamic.

The key concept to understand here is the so-called physiological complexity (or complex variability in physiology), an analytical approach to electrophysiological signal analysis stemming from electrical engineering, statistical physics and complex systems dynamics theory (chaos) (36–40). A more familiar name for this approach is fractal and nonlinear analysis (41). Despite the fact that many medical professionals are labeling this approach “novel” it is not novel by any standard; seminal work by Mandelbrot from 70's, Pincus, Hausdorf, Peng, and Goldeberger from 80's and 90's last century made that possible. They all built on early mathematics work of Cantor (Cantor's set, 1893), Peano (1890), Sierpinski (Triangle,1907), Koch (Snowflake, 1909), Lucia (Lucia's set, 1917) and others who could not generalize their findings before the advent of modern computers.

If a researcher in any medical field wants to explore the effect of a certain factor, the most probable way to do it is to calculate the means, standard deviations, *p*-values, and other measures coming from frequentist statistics that dominates the field. Irregularity statistics, like any entropy-based measure, for example, quantify the changes in physiological systems in a much more accurate and practically meaningful way (42). These two approaches (standard or conventional vs. non-linear) are simply measuring different information contained in the data, but as repeatedly shown physiological signals are far more complicated that we previously thought (43). Knowing that human physiology is not linear (in essence, not additive) and has many fractal dependencies in its control mechanisms, a better approach to analyzing signals from such a complex systems dynamics would be non-linear analysis (38, 39). Wouldn't it be logical to apply analytics that is better suited for non-stationary, non-linear, and noisy signals, than to just focus on how smeared are the data around the means?

From the existing literature, coming mainly from engineering and technical background, it is clear that fractal and non-linear analysis is much better suited for this task (38, 41).

Just to mention some of the facts important in research of depression (and mood disorders in general), to improve the understanding of the above-mentioned research. Cortico-vagal control (CVC) is connected to heart rate variability (HRV), which showed to be a robust marker of depression, anxiety, and several other psychiatric conditions (44–46). Cortico-vagal control (as well as many structural and functional physiological phenomena) is proven to have fractal nature (40, 44). Heart dynamics also has a fractal structure (40). There is much research evidence on the connection between autonomous nervous system (ANS) and heart dynamics in depression, obtained by use of non-linear analysis approach (47–51). There is also evidence that non-linear measures are much more effective in detecting this relationship with a much larger effect size in publications in the last two decades [(52) in review].

What we know now, from the analytical perspective and possible application in clinical practice, is that by the mere addition of that specific non-linear characterization of signal, possible in real-time, one can: detect depression (10, 11, 34, 53, 54), detect the subtypes of depression—melancholic vs. non-melancholic (49), detect comorbidities (48), discern episode and remission phase (9), detect cardiovascular risks early (55), differentiate between unipolar and bipolar depression (56) and even detect existing but unreported suicidal ideation (57). As we already know that small sample sizes jeopardize the overall accuracy of the ML models, the only solution to generalize and effectively arrive at real-life translation to clinical practice of those promising methods of detection/classification is to *collect more data*. The only way to go, is to organize large collaborative projects with identical protocols of data collection, similar to STAR^*^D. Like many things in life we should try to keep it simple: base our research on already successful research based on small samples, but increase the size of a sample; add some form of electrophysiological data and non-linear feature extraction; keep dimensionality of a problem as low as possible; always perform external validation and once we deploy the model developed in lab, keep monitoring its performance. In order to make the research reproducible, we might preregister the protocols and methods, and publish our negative results. Collaborative data sharing (anonimizied data are a good practice but time series required here are already GDPR compliant) can also contribute to the solution of this problem.

With todays' technology that made possible Telehealth &IoT (portable monitoring devices, with medical-grade signal quality), as a reliable way of remote monitoring of outpatients, we can support clinicians with objective additional information that might largely improve the effectiveness of therapy for depression. It might be close to previously envisaged personal medicine, increasing the ability of every clinician to better navigate many diagnostic decisions. Revisiting some not-so-well-known mathematical concepts that can thrive with cloud technology, would pay off in improved psychiatric diagnostics and treatment.

Although the citation is coming from the economy, it effectively applies to the adoption of these innovations in psychiatry: *The difficulty lies not in the absence of new ideas, but an escape from the old ones* (58).

## Author Contributions

MČ envisaged and designed the paper and performed a literature search. MČ and VL wrote the paper, reviewed the paper, and corrected the text. All authors contributed to the article and approved the submitted version.

## Funding

This work was partially supported by the grant PID2020-113192GB-I00 (Mathematical Visualization: Foundations, Algorithms, and Applications) from the Spanish MICINN.

## Conflict of Interest

MČ was employed by 3EGA B.V. The remaining author declares that the research was conducted in the absence of any commercial or financial relationships that could be construed as a potential conflict of interest.

## Publisher's Note

All claims expressed in this article are solely those of the authors and do not necessarily represent those of their affiliated organizations, or those of the publisher, the editors and the reviewers. Any product that may be evaluated in this article, or claim that may be made by its manufacturer, is not guaranteed or endorsed by the publisher.
